# The Largely Normal Response to Toll-Like Receptor 7 and 9 Stimulation and the Enhanced Expression of SIGIRR by B Cells in Systemic Lupus Erythematosus

**DOI:** 10.1371/journal.pone.0044131

**Published:** 2012-08-29

**Authors:** Yun-Yan Zhu, Yin Su, Zhan-Guo Li, Yu Zhang

**Affiliations:** 1 Department of Immunology, and Key Laboratory of Medical Immunology of Ministry of Health, Peking University Health Science Center, Beijing, China; 2 Department of Rheumatology and Immunology, Peking University People’s Hospital, Beijing, China; Institute of Zoology, Chinese Academy of Sciences, China

## Abstract

**Background:**

Altered Toll-like receptor (TLR) signaling has been implicated in the pathogenesis of systemic lupus erythematosus (SLE). The present study was undertaken to characterize responses of B cells from SLE patients to TLR7 and TLR9 stimulation and to explore the potential role of single immunoglobulin interleukin-1 receptor related molecule (SIGIRR) in the regulation of TLR-mediated responses of SLE B cells.

**Methodology/Principal Findings:**

Peripheral blood mononuclear cells (PBMC) were isolated from 64 patients with SLE and 37 healthy donors. CD19+ B cells purified using microbeads were cultured with TLR7 or TLR9 agonists. Cell proliferation was measured by thymine incorporation and the frequency of antibody-secreting cells was determined by ELISPOT assay. SIGIRR expression in PBMCs and B cells was analyzed using flow cytometry analysis. In contrast to the enhanced proliferation following B cell receptor (BCR) engagement, B cells from SLE patients exhibited a virtually normal proliferative response to TLR7 or TLR9 stimulation. Moreover, B cells from SLE patients and healthy donors were almost equally competent to differentiate into antibody-secreting cells upon TLR engagement except for a reduction in the generation of IgG-secreting cells by TLR9-stimulated lupus B cells. In line with these somehow unexpected observations, SLE B cells were found to express a significantly higher level of SIGIRR than normal B cells.

**Conclusions/Significance:**

Despite the reported upregulation of TLR7 and TLR9 expression in B cell from SLE patients, their responses to TLR stimulation were largely normal. The increased expression of the negative regulator SIGIRR may be partly responsible for the “balance of terror”.

## Introduction

Systemic lupus erythematosus (SLE) is a prototypic autoimmune disease affecting multiple tissues and organs with a diverse array of clinical manifestations. Among the wide variety of immunological aberrations associated with SLE, most prominent is the presence of auto-reactive T and B cells with specificity for self molecules commonly found in the nucleus, such as double-stranded DNA (dsDNA) and RNA-containing small nuclear ribonucleoprotein (snRNPs) [1]. While T cell has long been considered as a major player in the pathogenesis of SLE, B cell abnormalities have received much attention in recent years, partly because of the remarkable success of B cell depletion as a treatment for SLE. Patients with active SLE have been found to have1.5–4-fold more IgG and IgM-secreting cells in the peripheral blood, with a concomitant increase in the number of B cells secreting autoantibodies, especially anti-DNA antibodies [2]. Moreover, B cells from SLE patients exhibit augmented calcium response and increased tyrosine phosphorylation upon BCR crosslinking [3].

The precise mechanisms underlying the altered B cell compartment in SLE remains elusive. There is increasing evidence, however, that TLR-mediated signals are critically involved in this process [4], [5]. TLRs are a group of receptors recognizing conserved molecular patterns expressed by exogenous pathogens or displayed on certain endogenous molecules. To date, 10 TLRs have been identified in the human genome, many of which are constitutively or inducibly expressed in human B cells [6], [7], [8]. Stimulation of B cells with TLR ligands not only leads to cell proliferation and antibody production and class switching, but also promotes the expression of co-stimulatory molecules and secretion of various cytokines, which presumably may contribute to the enhanced capacity of B cells as antigen-presenting cells [4]. Data supporting the involvement of TLRs in autoimmunity mainly come from studies using murine lupus models. A pioneering study by Marshak-Rothstein’s group demonstrated that effective activation of transgenic B cells expressing antigen receptor specific for IgG2a (AM14 B cells) was only induced by IgG2a-chromatin immune complexes and requires the synergistic engagement of BCR and TLR9 [9]. Similarly, the activation of AM14 B cells by RNA and RNA containing auto-antigens was achieved only upon dual engagement of BCR and TLR7 [10]. In both cases, BCR is believed to facilitate the delivery of nucleic acids to TLR containing endosomal compartments. Consistent with the *in vitro* finding, *in vivo* results with *Tlr7*-deficient lupus-prone mice verify the pathological significance of TLR7 in SLE. In the absence of *Tlr7*, the generation of autoantibodies to RNA-containing antigens was greatly impaired and the clinical diseases were much ameliorated [11], [12]. Analysis of the Y-linked autoimmune accelerator (*Yaa*) locus renders further support for such a point. *Yaa* is known to be a genetic modifier capable of increasing the severity of SLE. Recent studies revealed that this locus contained a duplication of *Tlr7* [13], [14], and the majority of the autoimmune phenotype associated with *Yaa* appeared to be conferred by the two-fold increase in TLR7 expression [15]. The *in vivo* effect of TLR9 on autoimmunity, on the other hand, is not fully congruent with expectations. In one initial study with the lupus model induced by anti-DNA BCR transgene and homozygous deficiency of the inhibitory receptor FcγIIB, lack of *Tlr9* was found to block class switching of autoreactive B cells to the pathogenic IgG2a and 2b subclasses with reduced pathology and mortality [16]. Subsequent studies with the more commonly used MRL/Mp^lpr/lpr^ model, however, revealed that TLR9 could represent a protective factor as its deficiency resulted in increased immune activation and accelerated lupus nephritis and mortality [12], [17]. Thus, TLR7 and TLR9 appear to have divergent effect on the development of SLE.

To avoid the potentially detrimental effect arising from inappropriate activation of TLRs, TLR signaling is normally under tight control by a number of negative regulators. They attenuate TLR signaling by acting as decoy receptors, interfering with the downstream signaling pathway, or facilitating the degradation of TLRs [18] **.** Among them, SIGIRR (also known as TIR8) is of particular interest for its close association with autoimmunity [19]. SIGIRR is a member of the IL-1R like receptor family, which is characterized by a single extracellular immunoglobulin domain and a cytoplasmic TIR domain missing two well conserved residues (Ser447 and Tyr536) essential for IL-1R signaling [20]. *In vitro* data demonstrate that enforced expression of SIGIRR inhibits IL-1R and TLR-induced NF-κB activation and cytokine production while lack of SIGIRR enhances the responses [21], [22], [23], [24], [25], [26], [27], [28]. *In vivo*, *Sigirr-*deficient C57BL/6^lpr/lpr^ mice develop severe systemic autoimmunity as indicated by massive lymphoproliferation, production of autoantibodies against numerous lupus autoantigens and autoimmue tissue injury. Moreover, dendritic cells (DCs) and B cells from such mice show much enhanced responses to immune complexes containing RNA and DNA antigens [24]. In another study, *Sigirr* deficiency is found to aggravate hydrocarbon oil-induced lupus, possibly due to hyperactivation of DCs by TLR7-mediated signal [29]. Therefore, *Sigirr* may represent a novel SLE susceptibility gene.

Despite the accumulating evidence that dysregulated TLR signaling contributes to the pathogenesis of lupus in animal models, limited information is currently available concerning its role in human diseases except for several recent reports on the upregulated expression of TLR7 and TLR9 in B cells from lupus patients [22], [30], [31], [32], [33]. The functional consequence of their elevated expression, however, remains poorly understood. Even less known is the regulation of TLR signaling in human B cells, either in physiological or pathological conditions. The present study was therefore undertaken to characterize the responses of B cells from SLE patients to TLR7 and TLR9 stimulation and to explore the potential role of SIGIRR in the regulation of TLR-mediated responses of SLE B cells.

## Methods

### Objectives

To characterize the responses of B cells from SLE patients to TLR7 and TLR9 stimulation and the expression of SIGIRR in SLE B cells.

### SLE Patients, Control Subjects and Blood Samples

Peripheral blood samples were obtained from 64 patients attending the rheumatology clinic at Peking University People’s Hospital. All the patients fullfilled the American College of Rheumatology 1982 criteria for SLE. Disease activity was assessed by the SLE Disease Activity Index (SLEDAI). Of the 64 patients, 52 had active disease (SLEDAI≥8) while the rest were inactive at the time of enrollment. Control samples from 31 healthy donors were provided by the blood bank of Peking University People’s Hospital. All patients had not taken any immunosuppressive therapy at least for 24 h before blood sample were collected.

### Reagents

F(ab’)_2_ fragments of goat anti-human IgG plus IgM plus IgA (H+L) used for BCR crosslinking were purchased from Jackson ImmunResearch Laboratories (West Grove, PA), human TLR9 ligand CpGODN2006 and TLR7/8 ligand CL097 from InvivoGen (San Diego, CA), rabbit anti-SIGIRR and goat anti-rabbit IgG (H+L)-FITC from Abcam (Cambridge, UK), CD19-Percp (HIB19) and isotype-control mouse IgG1,κ (MOPC-21) from Biolegend (San Diego, CA).

### B Cell Isolation and Culture

PBMCs from SLE patients and healthy donors were isolated by Ficoll-Hypaque density gradient centrifugation of EDTA-anticoagulated blood samples. B lymphocytes were isolated from PBMCs using CD19 microbeads (Miltenyi Biotec, Bergisch Gladbach, Germany) according to the instruction of the manufacturer. The purity of CD19^+^ cell was more than 95% as analyzed by flow cytometry. Purified B cells were cultured in round-bottom 96-well tissue culture plates (Nalge Nunc, Rochester, NY) in Opti-MEM (Invitrogen, San Diego, CA) supplemented with 10% heat-inactivated fetal calf serum (FCS) (Hyclone, Logan, UT) and 200 U/ml gentamycin. CpGODN2006, CL097 and F(ab’)_2_ fragments of goat anti-human IgA plus IgG plus IgM (H+L) were added to the culture at the concentration as specified in the **Results**.

### Cell Proliferation Assay

Cell proliferation was analyzed by [^3^H] thymidine incorporation. Purified B cells were cultured in 96-well plates at 1.5×10^5^cells/ml in 200 µl medium under various conditions for 72 h, and [^3^H] thymidine (0.5 µCi/well) was added into the culture in the last 8 h. Cells were then harvested, and the thymidine incorporation was measured using a β-scintillation counter (Beckman, Fullerton, CA).

### Enzyme Linked Immunospot (ELISPOT) Assay

The frequency of IgM- and IgG-secreting cells was determined by ELISPOT assay. Briefly, B cells were first exposed to various stimulators for 96 h, and replated at 1.5×10^4^/ml into 96-well nitrocellulose plates (Millipore, Bedford, MA), which were precoated with 5 µg/ml AffiniPure goat anti-human IgG (H+L) (Jackson ImmunResearch Laboratories) or goat anti-human IgM (μ chain specific) (Sigma-Adrich, St. Louis, MO) and blocked with Opti-MEM medium containing 10% FCS. After culturing for another 24 h, the cells were washed off. The plates were subsequently incubated with biotin-labeled goat F(ab’)_2_ anti-human IgM (μ chain specific) (used at a dilution of 1∶8000) or anti-human IgG (γ chain specific) (used at a dilution of 1∶6000) (Southern Biotech, Birmingham, AL) for 2 h at 37°C, followed by incubation with streptavidin-alkaline phosphatase (Mabtech, Nacka, Sweden). The plates were developed using 5-bromo-4-chloro-3-indolyl phosphateynitroblue tetrazolium (Sigma-Aldrich) as a chromogenic substrate. After the final wash, the spots on the plate membrane were counted using an ELISPOT reader (Sage Creation, Beijing, China). The average spots in duplicated wells were calculated and expressed as the number of antibody-secreting B cells.

### Flow Cytometric Analysis

Flow cytometric analysis was performed to measure SIGIRR expression on B cell surface. PBMCs were first incubated with rabbit polyclonal antibodies against SIGIRR (1 µg per 10^6^ cells), followed by staining with goat anti-Rabbit IgG (H+L)-FITC (the secondary antibody) and anti-CD19-PerCP. Data acquisition and analysis was performed on FACSCalibur using the Cellquest software (BD Biosciences). The expression level of SIGIRR on CD19^+^ cells was converted to relative fluorescence index (rFI) as follows: rFI = [Geometric Mean Fluorescence Intensity (Geo MFI) with anti-SIGIRR - Geo MFI with secondary antibody alone]/Geo MFI with secondary antibody alone.

### Ethics

This study was approved by the Ethics Committee of Peking University People’s Hospital. All patients involved gave written informed consent prior to study enrollment according to the principles expressed in the Declaration of Helsinki.

### Statistical Analysis

Data were presented as mean ± standard deviation. The statistical significance of the difference between groups and correlations between SIGIRR expression and clinical data of SLE patients was determined by unpaired t test and Pearson’s correlation test, respectively, using GraphPad Prism software (GraphPad, La Jolla, CA). Differences were considered significant when p values were less than 0.05.

## Results

### Demographic and Clinical Laboratory Data of Patients with SLE

A total of 64 SLE patients were enrolled in the present study. The demographic and clinical laboratory data of these patients were abstracted from hospital records and summarized in Table 1. Due to the limitation in the amount of peripheral blood samples available, the 64 patients were divided into three sets randomly : Set 1 with 18 patients were analyzed for cell proliferation; Set 2 with 17 patients for frequency of IgG- and IgM-secreting cells; Set 3 with 29 patients for SIGIRR expression.

**Table 1:** Clinical characteristics of SLE patients studied.

Items	Set 1	Set 2	Set 3
Number of patients	18	17	29
Age, years	40.91±14.61	37.11±15.41	36.61±15.58
Sex, male/female	3/15	3/14	6/23
Duration of diagnosis, months, mean (range)	66.54 (2–312)	66.88 (3–384)	71.21 (1–360)
Anti-dsDNA −/+	7/11	7/7*	8/21
ANA−/+	1/17	1/16	5/24
C3(g/L)	0.51±0.23	0.55±0.21	0.54±0.22
C4(g/L)	0.12±0.12	0.10±0.10	0.12±0.09
ESR(mm/h)	31.08±24.83	31.33±25.19	27.51±20.51
CRP(mg/L)	3.66±5.25	8.65±20.99	9.84±18.78
IgA(g/L)	3.09±2.23	3.20±1.45	2.90±1.37
IgG(g/L)	13.57±3.97	13.85±4.01	12.63±4.65
IgM(g/L)	1.30±0.83	1.13±0.61	0.93±0.51
SLEDAI<8/> = 8	5/13	0/17	7/22

Values are expressed as mean ± standard deviation (s.d.), unless stated.

* anti-dsDNA reactivity was not determined for 3 patients in Set 2.

### The Largely Normal Proliferative Responses of SLE B Cells to TLR7 and TLR9 Signaling

**Figure 1:**
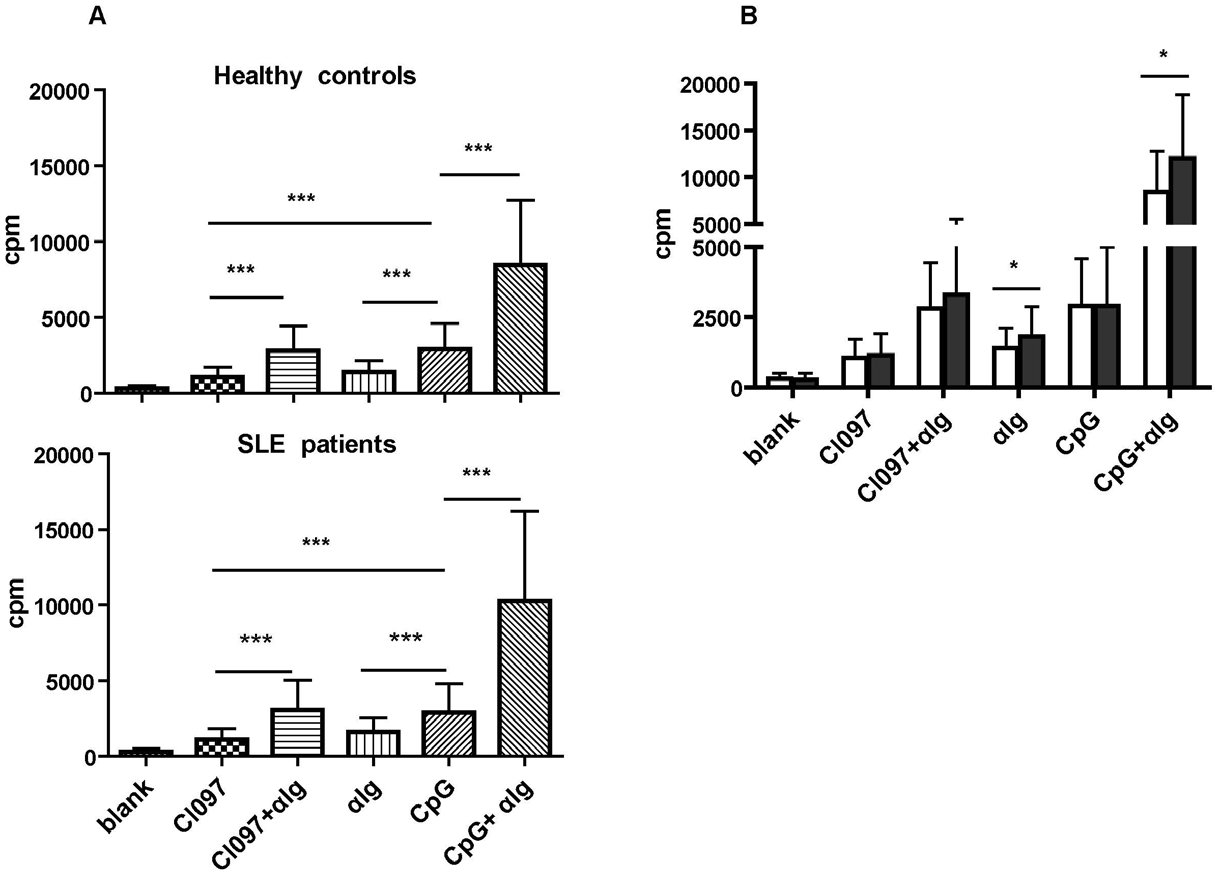
Proliferation of B cells from SLE patients and healthy controls following TLR and/or BCR ligation. Purified CD19^+^ B cells from SLE patients (n = 18) and healthy controls (n = 14) were cultured for 72 h in medium alone or in the presence of CL097 (2.5 µg/ml), CpGODN2006 (1 µM), anti-Ig(2.5 µg/ml), CL097+anti-Ig, or CpGODN2006+anti-Ig. Cell proliferation was measured by thymidine incorporation. A. Thymidine incorporation by B cells from healthy controls (upper panel) and SLE patients (lower panel) in response to the various stimuli. B. Direct comparison of the proliferative response of SLE B cells (filled bars) with that of control B cells (open bars). Data are presented as mean ± SD. Statistical significance was tested by non-paired t test. *P<0.05, ***P<0.0001.

Increased levels of TLR7 and TLR9 expression have been repeatedly documented in peripheral B cells from SLE patients [30], [31], [32], [33], [34]. We sought to determine whether the enhanced receptor expression was associated with alterations in cell responses to the cognate ligands. To this end, CD19+ B cells were isolated from the peripheral blood of 18 lupus patients and 14 healthy donors, and compared for their proliferative capacity following stimulation with the TLR7 agonist CL097 and the TLR9 agonist CpGODN2006. As expected, substantial proliferation of B cells was induced by CpGODN2006 and, to a less extend, by CL097 (Figure 1A). But B cells from SLE patients and healthy donors showed equal levels of thymidine incorporation, which were in contrast to the significantly enhanced proliferation of SLE versus normal B cells in response to BCR engagement (Figure 1B). Further, we tested potential synergistic effects of BCR- and TLR-mediated signals in B cell activation. In accordance with previous reports [8], [35], simultaneous stimulation of BCR and TLR9 induced an increase in thymidine incorporation, which was apparently more prominent than simple addition of the effects induced by each signal alone. On the other hand, the extent of increase following co-ligation of BCR and TLR7 appeared to be more consistent with an additive effect (Figure 1A). Again, there was no major difference between B cells from SLE patients and those from healthy donors except for the slightly increased response of SLE B cells to the co-stimulation of BCR and TLR9, which was most likely due to the augmented BCR signaling in these cells (Figure 1B). These results indicate that SLE B cells are comparable with normal B cells in their proliferative response to TLR stimulation, either by itself or in combination with BCR stimulation.

### Impaired Formation of IgG-secreting Cells by SLE B Cells Stimulated with TLR9 Agonists

**Figure 2:**
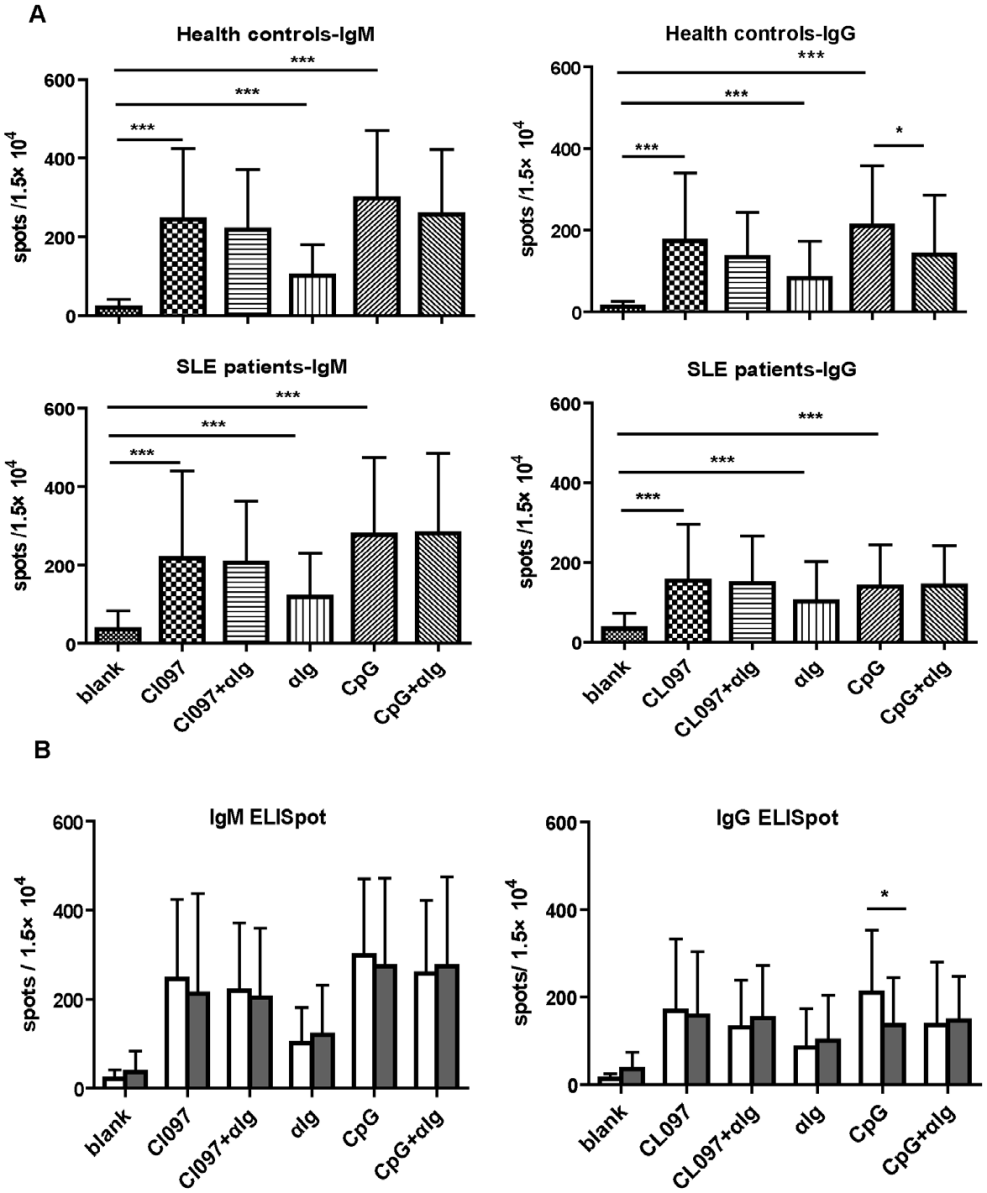
Frequency of antibody secreting cells among SLE B cells following TLR and/or BCR ligation. CD19^+^ B cells purified from SLE patients (n = 17) and healthy controls (n = 17) were cultured for 96 h in medium alone or in the presence of CL097 (2.5 µg/ml), CpGODN2006 (1 µM), anti-Ig(2.5 µg/ml), CL097+anti-Ig, or CpGODN2006+anti-Ig. The frequency of IgM- or IgG-secreting cells was analyzed by ELISPOT assay. A. The number of IgM- or IgG-secreting cells for each of 1.5×10^4^ SLE or control B cells generated under the various conditions. B. Direct comparison of SLE B cells (filled bars) with control B cells (open bars) for their capacity to generate IgM- or IgG-secreting cells. Data are present as means ± SD. Statistical significance was tested by non-paired *t* test. *P<0.05, ***P<0.0001.

TLR7 and TLR9 signaling in B cells culminates in plasma cell differentiation and antibody secretion [6]. We next explored whether B cells from SLE patients would be different from their normal counterparts in the generation of antibody-secreting cells upon TLR stimulation. Purified B cells from SLE patients and healthy controls (n = 17 for each group) were cultured in the presence of TLR7 or TLR9 agonists, and the frequency of IgM- or IgG-secreting cells was determined using ELISPOT assay. As shown in Figure 2A, CpGODN2006 and CL097 at the tested concentrations displayed equal potency in driving the differentiation of B cells into antibody-secreting cells. Moreover, comparable results were obtained with SLE and control B cells in most cases. In fact, the number of IgG-secreting cells induced by CpGODN2006 was even reduced in SLE B cells (Figure 2B).

In contrast to the synergistic effect on cell proliferation, continuous BCR signaling has been reported to inhibit TLR9-induced plasma cell differentiation of murine B cells via ERK activation [35]. Similar inhibition was observed for the differentiation of CpGODN-stimulated human B cells into IgG-secreting cells but not into IgM-secreting cells. Intriguingly, such an antagonistic effect was lost in B cells from SLE patients (Figure 2A), suggesting a potential defect in SLE B cells in the inhibitory mechanism which prevented excessive immunoglobulin synthesis.

### Increased SIGIRR Expression in B Cells from SLE Patients

**Figure 3:**
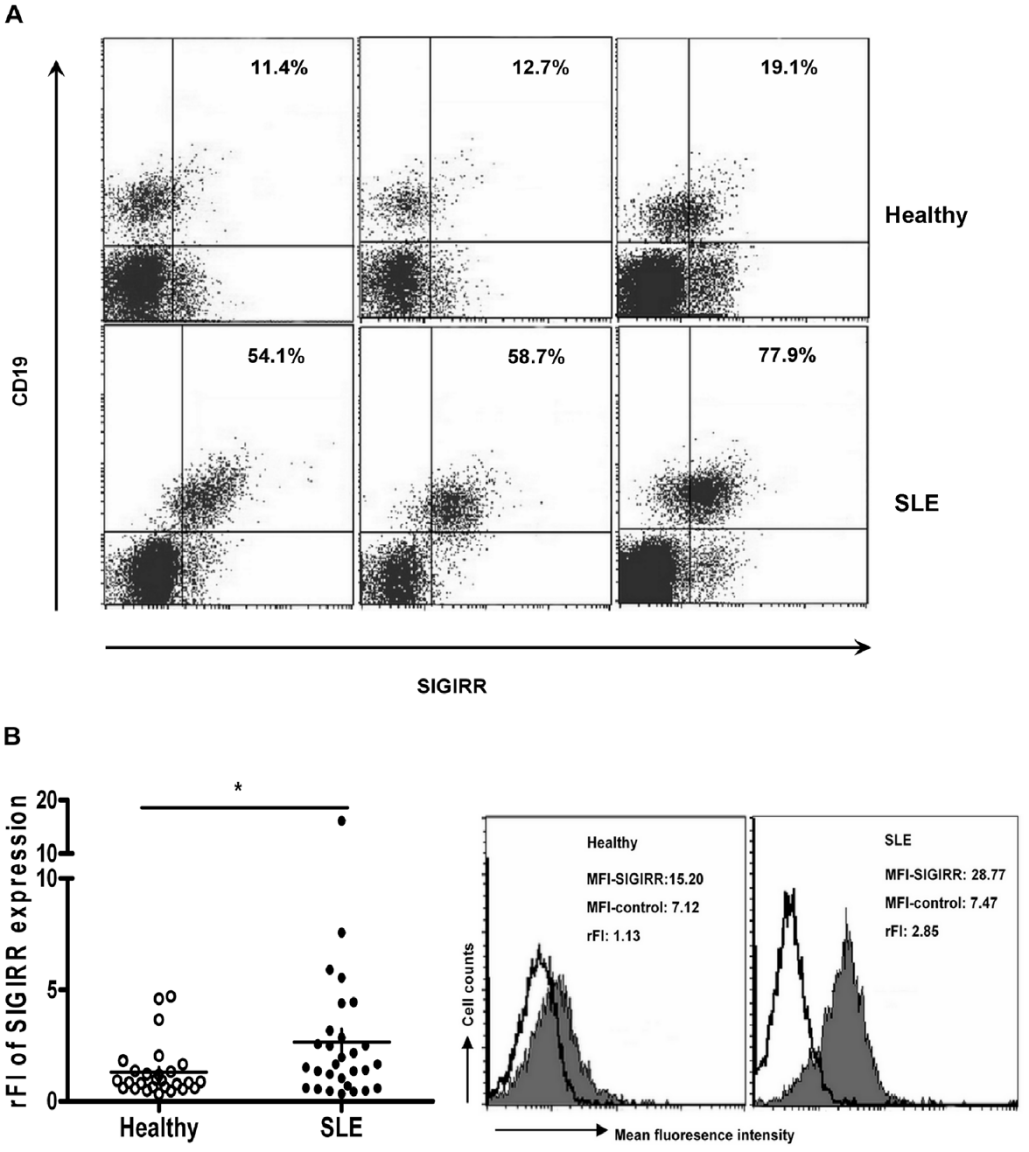
SIGIRR expression by B cells from SLE patients (n = 29) and healthy controls (n = 27). SIGIRR expression was analyzed by flow cytometry following staining with anti-CD19 and anti-SIGIRR antibodies. A. The staining profile of representative samples from SLE patients and healthy subjects, three for each. The numbers indicate the percentage of CD19^+^SIGIRR^+^ cells. B. The expression levels of SIGIRR in SLE and control B cells as measured by rFI. The histograms on the right show SIGIRR expression (shaded area) on CD19^+^ cells from a representative SLE patient and a representative healthy subject. The black line represents a control with FITC-labeled secondary antibody alone. The inserted numbers indicates MFI with anti-SIGIRR antibody (MFI-SIGIRR), MFI with secondary antibody alone (MFI-control), and the deduced rFI. The panel on the left shows the rFI value for each individual subjects. The horizontal bar denotes the mean. Statistical significance was tested by non-paired *t* test. *P<0.05.

Given the reported upregulation of TLR7 and TLR9 expression in B cells from SLE patients [30], [31], [32], [33], [34], it was surprising to see that these cells mounted virtually normal, and sometimes even reduced responses to the stimulation of the corresponding ligands. These seemingly contradictory findings prompted us to examine the expression of negative regulators for TLR signaling. The current study was focused on SIGIRR as its deficiency has been shown to lead to the development of lupus-like syndrome in the mouse [24]. PBMCs were isolated from 29 SLE patients and 27 healthy donors, and stained for surface expression of SIGIRR and CD19. While a SIGIRR^+^ subset was readily distinguishable in the CD19^−^ population, SIGIRR expression in CD19^+^ cells appeared to be more homogenous (Figure 3A). Using an arbitrary gate set according to SIGIRR expression in the CD19^−^ population, we saw that the CD19^+^ population in SLE patients usually contained more SIGIRR^+^ cells (Figure 3A). To more accurately depict SIGIRR expression in B cells, the raw data obtained from flow cytometry were converted into relative fluorescence index (rFI). As shown in Figure 3B, the rFI value was significantly higher for B cells from SLE patients than those from healthy donors, indicating an overall increase of SIGIRR expression in SLE B cells.

**Figure 4:**
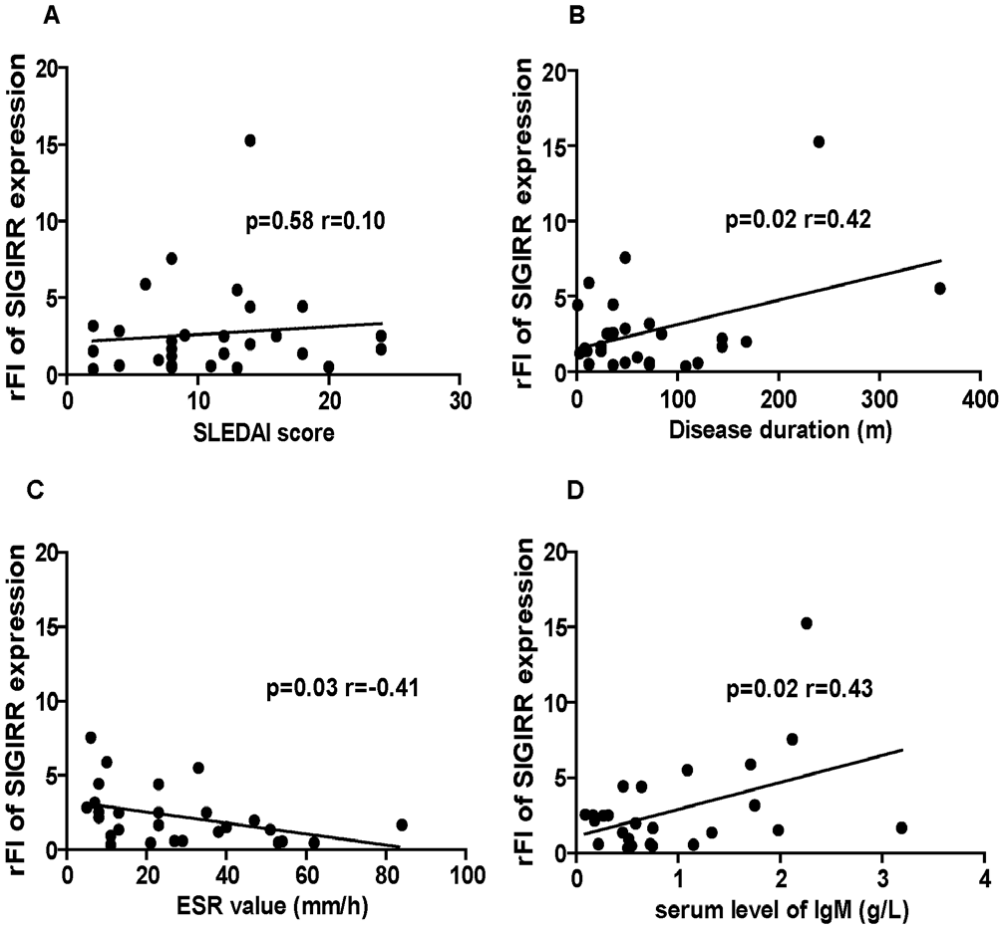
Correlation of SIGIRR expression and clinical features. The correlation between the SIGIRR expression level in B cells and the clinical features of SLE patients, including SLEDAI score (A), disease duration (B), ESR value (C) and serum IgM (D) was analyzed by Pearson’s correlation test. The inserted numbers show the *p* and *r* values.

Subsequently, we analyzed the potential correlation between the expression levels of SIGIRR in B cells and the clinical features of SLE patients. A statistically significant correlation was observed with the serum levels of IgM antibody (*p* = 0.02, *r* = 0.43) and with disease duration of SLE patients (*p* = 0.02 *r* = 0.42). In addition, SIGIRR expression was found to be inversely correlated with ESR (*p* = 0.03, *r* = −0.41) (Figure 4). No correlation, however, was revealed between SIGIRR expression and SLE disease activity (Figure 4), and other parameters, including total serum IgG or IgA, anti-dsDNA, anti-nuclear antibodies, C3, C4, and CRP (data not shown).

## Discussion

Several recent studies, using either flow cytometry or RT-PCR, have detected increased expression of TLR9 in CD19^+^ or CD20^+^ B cells from SLE patients [30], [32], [33], [34]. An additional study has documented higher levels of mRNA for both TLR9 and TLR7 in PBMCs [31]. However, it remains to be determined whether the upregulation of TLR9 and TLR7 would lead to altered B cell responses to the cognate ligands. The present study was focused on two major aspects of B cell activation, namely cell proliferation and antibody production. As previously reported [6], human B cells proliferated vigorously in response to CpGODN stimulation whereas CL097 induced only minimal proliferation. Nevertheless, B cells from SLE patients and healthy donors showed no difference in their proliferative response to either CpGODN or CL097. As for antibody production, B cells from the two groups yielded similar numbers of IgM- or IgG-secreting cells when treated with the TLR7 agonist. Under TLR9 stimulation, however, IgG-secreting cells were found to be reduced for SLE B cells even though IgM-secreting cells remained comparable. These results are apparently contradictory to the prediction that increased receptor expression would result in augmented responses to the ligands. Moreover, a few previous studies did show enhanced activation of SLE B cells as measured by certain parameters. Nakano et al., for example, reported that TLR9 engagement led to the production of anti-dsDNA antibody and IL-10 in SLE B cells but not in normal B cells [32]. Another study demonstrated that the upregulation of TLR9 expression was associated with enhanced induction of HLA-DR in lupus B cells [30]. Still another study, however, revealed comparable TLR9-induced secretion of IgM and IL-10 by PBMCs from lupus patients and healthy donors [36]. There can be multiple reasons for this discrepancy. Firstly, different aspects of B cell activation, such as proliferation, antibody production, cytokine secretion and expression of co-stimulatory molecules, may be differentially regulated by TLR signals in SLE patients. Secondly, PBMCs versus purified B cells were used in some of the studies, which raise the possibility that the altered B cell response may actually be a secondary effect. Lastly, the discrepancy may result from the considerable variations in the subjects under investigation, including sample size, clinical features and medical treatment. Using purified B cell from a relative large cohort, the present study demonstrates that SLE B cells mount a largely normal, if not diminished, response to TLR7 and TLR9 signaling in terms of cell proliferation and antibody production.

In addition to single treatment with the TLR7 or TLR9 agonist, we examined the responses of SLE B cell to simultaneous stimulation of TLR and BCR. The BCR-mediated signal has been shown to synergize with TLR9 signal to promote B cell proliferation, possibly through upregulating TLR9 expression [8], [35]. In contrast, continuous BCR signaling has a profound inhibitory effect in the formation of antibody-secreting cells induced by TLR9 engagement [35]. We confirmed the synergistic effect of BCR and TLR9 signals in the proliferation of B cells, whereas no obvious synergism was seen between TLR7 and BCR signals. As much as the antibody response is concerned, BCR crosslinking indeed resulted in the suppression of TLR9-induced generation of IgG-secreting cells from normal B cells. Notably, such an inhibitory effect was lost in SLE B cells. This result is reminiscent of several previous reports in which B cells from lupus-prone mice or SLE patients were found to be resistant to anti-BCR-mediated inhibition of either LPS-induced IgM production [37] or PWM-induced activation [38]. The pathological relevance of the seemingly general loss of the antagonism mediated by BCR in SLE B cells certainly warrants further investigation.

TLR signaling is tightly controlled by a number of negative regulators, including SIGIRR [18], [19]. Recent studies with knockout mice revealed a non-redundant anti-inflammatory function of this molecule in various conditions. In dextran sulfate sodium-induced colitis model, for example, SIGIRR deficiency leads to much more severe intestinal inflammation in terms of weight loss, intestinal bleeding, local tissue damage and mortality [26], [39]. In addition, *Sigirr*-deficient animals exhibit exacerbated Th2 responses in OVA-challenged asthma model [40] and joint inflammation in collagen antibody-induced arthritis model [27]. Notably, lack of SIGIRR in C57BL/6^lpr/lpr^ background causes massive lymphoproliferation, production of autoantibodies against numerous lupus autoantigens and autoimmue tissue injury, possibly due to enhanced responses of DCs and B cells to immune complexes containing RNA and DNA antigens [24]. To elucidate the potential role of SIGIRR in the human disease, we analyzed its expression in PBMCs from patients with SLE. While there is considerable variation in the expression levels in the non-B cell fraction, B cells from SLE patients generally show enhanced SIGIRR expression. Although the pathological significance of such an increase is not clear, it provides an explanation for the contradiction of the reported upregualtion of TLR7 and TLR9 in but largely normal or even diminished responses to the corresponding ligands for SLE B cells. Due to the limited volume of blood sample that could be possibly obtained from each individual patient, we were unable to perform simultaneous analysis of expression levels of TLR7, TLR9 and SIGIRR and the proliferative and antibody responses. Further studies are needed to clarify whether there are direct correlations among these parameters.

The mechanism underlying the upregulation of SIGIRR expression in SLE B cells remains to be determined. In addition to epithelial tissues [20], [21], [23], [26], SIGIRR is found to be expressed in various types of immune cells, including monocytes [25], [26], [27], [41], DCs [26], [27], NK cells [21], T cells [40], [42], and B cells [43]. Importantly, the status of cell differentiation and activation, in a cell type-specific manner, has a profound impact on SIGIRR expression. In epithelial cells, LPS treatment usually leads to the downregulation of SIGIRR transcripts [21], [23], [25], whereas stimulation of resident intrarenal myeloid cells with LPS, tumor necrosis factor or interferon exhibits an opposite effect [25]. Similarly, monocyte differentiation into macrophage and DC maturation is accompanied with a reduction in SIGIRR expression [26], [27] while its expression is increased during Th17 and Th2 cell development [40], [42]. In the latter scenario, loss of function of SIGIRR results in enhanced polarization to Th17 and Th2 cells. This, as well as the finding of elevated levels of SIGIRR in monocytes from patients with sepsis or non-infectious systemic inflammatory response [41], leads to the proposal that SIGIRR provides a feedback regulatory mechanism. It is tempting to speculate that a similar mechanism is activated in B cells from SLE patients to prevent excessive B cell response to the TLR signal.

In summary, the present study demonstrated that, despite the reported upregualtion of TLR7 and TLR9, B cells from SLE patients mounted a largely normal, if not diminished, response to the TLR signal in terms of cell proliferation and antibody secretion. This contradiction may be partly explained by the elevated levels of SIGIRR expression. Intervention targeting the TLRs in SLE should take into consideration of the complicated network regulating the signaling pathway.
